# The polymorphism of rs266729 in adiponectin gene and type 2 diabetes mellitus: A Meta-Analysis

**DOI:** 10.1097/MD.0000000000008745

**Published:** 2017-11-27

**Authors:** Panpan Sun, Li Liu, Jiaxin Chen, Yuansi Chen, Litong Shi, Mustapha Umar Imam, Yanzi Chen, Xiaoting Pei, Yiping Xu, Yaxin Guo, Zhiguang Ping, Xiaoli Fu

**Affiliations:** a Department of Biostatistics and Epidemiology, College of Public Health; b Department of Histology and Embryology, Basic Medical College, Zhengzhou University, Zhengzhou, Henan, China.

**Keywords:** adiponectin gene, genetic models, rs266729, type 2 diabetes mellitus

## Abstract

Genome-wide association studies and meta-analyses indicate that the polymorphism of rs266729 in adiponectin gene increases the risk of type 2 diabetes mellitus (T2DM); however, these study methods have not been able to identify the underlying genetic effect on the development of T2DM. A genetic model-free approach was conducted to determine the underlying genetic model of inheritance of T2DM because of rs266729 in adiponectin gene.

We searched available studies on the association between the rs266729 in adiponectin gene and T2DM in accordance with the inclusion and exclusion criteria. Based on the information extracted from the studies, generalized odds ratio value (GOR) was used to evaluate whether the rs266729 polymorphism was a risk factor for T2DM. The parameter *λ* was calculated to estimate the genetic model, which was defined as the quotient of natural logarithm odds ratio of GC to CC divided by the natural logarithm odds ratio of GG to CC. Finally, binary logistic regression analysis was used to calculate the genetic effect of rs266729 on T2DM.

Data from 7 studies were included in this meta-analysis. The total number of subjects was 12,323, comprising 5,948 cases and 6,395 controls. Mean (standard deviation) age of cases was 59.50 (11.53), and that of the controls was 53.80 (11.65), whereas the proportion of male was 40.9 and 50.0%, respectively. GOR was 1.13 (1.02, 1.25) and *λ* was 0.47 (0.29, 0.64). The result of logistic regression indicated that the G allele influenced the development of T2DM in the additive model, whereas the genetic effect was 1.13 (1.06, 1.19). Sources of control populations were the cause of between-study heterogeneity; nonetheless, there was no publication bias among studies.

The G allele of rs266729 in adiponectin gene increases the risk of T2DM through an additive genetic model with an effect of 1.13 (1.06, 1.19).

## 1 Introduction

Diabetes mellitus (DM, ICD-10: E10.900, E11.900) is the most prevalent metabolic disorder that is characterized by chronic sustained hyperglycemia due to defects in insulin secretion from beta cells of the pancreas or resistance against insulin action. Its incidence has been increasing globally at an extremely rapid rate. In 2015, there were 415 million adults living with DM, and the number is expected to rise to 642 million by 2040. Type 2 diabetes mellitus (T2DM, ICD-10: E11.900) is the most prevalent form of DM and the health consequences and economic burden caused by this disorder are enormous.^[1]^ Reliable diagnostic tests for T2DM include the fasting blood glucose, oral glucose tolerance test, and glycated hemoglobin. However, there are still limitations with the use of these tools for the diagnostic screening of T2DM, and as such additional diagnostic biomarkers of T2DM are still being of explored.

The human adiponectin gene encoding the adiponectin protein,^[2]^ is negatively correlated with fasting glucose, insulin-resistance (IR), and T2DM.^[3–6]^ Epigenetic modifications, such as DNA methylation, of the adiponectin gene affecting gene transcription and health status, may be involved in T2DM.^[7]^ The methylation in the promoter region of this gene may result in repressed transcriptional activity of the adiponectin gene and low plasma adiponectin levels, leading to pathogenesis and progression of T2DM. Similarly, single-nucleotide polymorphisms (SNPs) and methylation in the promoter region of the gene could together affect adiponectin gene transcription. Genome-wide association studies (GWAS) have been used to evaluate the associations between the adiponectin gene promoter polymorphism, especially the rs266729, and T2DM.^[8,9]^ However, limitations of these studies have included: first, the reduction of the problem to a single comparison, either by performing 2 separate pairwise comparisons or by assuming a specific underlying genetic model. This method of multicomparisons declines statistical power, increases type I error rate, and false-positive rate. Second, the lack of insights into the association between the SNPs and genetic risks of developing DM, when such risks are controversial. For resolving the problem above, generalized odds ratio (GOR) was used in this study to evaluate the association between adiponectin rs266729 polymorphisms and disease status (T2DM vs. control) under complete genotype distribution, providing a summary risk effect and heterogeneity metric. Then a genetic model-free approach was conducted to estimate the rs266729 genetic effect on the development of T2DM and the underlying genetic model of inheritance, which may provide potential biomarkers and important evidence to establish prevention and treatment strategies for T2DM.

## 2 Methods

### 2.1 Literature sources

#### 2.1.1 Literature search

Potential eligible studies were identified by systematically searching the ISI Web of Science, Pubmed, MEDLINE, Elsevier, Springer, EBSCO, and Chinese Biological Medicine, China National Knowledge Infrastructure (CNKI), and Wanfang databases up to October 2016 using the terms: “adiponectin” “type 2 diabetes” “gene promoter region” and various synonyms. Search strategy: (“Adiponectin”) AND (“rs266729” OR “SNP-11377C/G” OR “SNP-11377” OR “C-11377G” OR “-11377C>G”) AND (“diabetes” OR “type 2 diabetes mellitus” OR “glycuresis” OR “fasting plasma glucose” OR “fasting blood glucose” OR “fasting blood sugar” OR “blood glucose level” OR “serum glucose”).

### 2.2 Inclusion and exclusion criteria

Studies that fulfilled the following criteria were included: studies showing the association between polymorphisms of adiponectin gene and T2DM; original articles of prospective cohort, case–control, or whole-genome association studies with raw data; studies with detailed criteria for T2DM diagnosis; studies showing the sample size of the 3 genotypes (CC, GC, GG) in both groups.

Studies were excluded if: they were not related to adiponectin rs266729 polymorphism; data were incomplete; when multiple published reports from the same population spanning the same time period are found, the one with the most useful information was included; they were series of studies, in which case the latest was included while the old ones excluded; sample size of any group was <500; family studies; genotype distribution of the control group deviated from Hardy-Weinberg equilibrium (HWE).

### 2.3 Data extraction and quality assessment

Data were independently extracted by 2 investigators (PS and LL) and checked by a third investigator (JC). Both investigators independently completed primary screening by reading the title and abstract of the studies, and subsequently screening the full article. The studies were included or excluded according to the criteria. The following information was extracted from all included studies: author, year of publication, continent, country, nationality, population, standards of T2DM diagnosis, average age and standard deviation, and frequencies of genotypes in both groups. If the 2 investigators had different opinions, consensus was reached through discussion. Emails were sent to corresponding authors for full texts where they were not available online. The quality of included studies in this meta-analysis was assessed by the Newcastle Ottawa Scale (NOS).^[10]^ A study was judged on 3 broad perspectives in the NOS scale: the selection of study groups (0–4 stars); comparability of groups (0–2 stars); ascertainment of the exposure of interest (0–3 stars). A higher score represented the better methodological quality; studies that received a rating of at least 7 stars were judged as “high quality.”

### 2.4 Statistical analysis

#### 2.4.1 The characteristic of included studies

HWE analysis for genotype distribution was checked with *χ*^2^ test in Excel 2007 (Microsoft Corporation). The significance level was set at 0.05, and all *P* values were 2-sided. This study was a meta-analysis, and ethical approval and informed consent were not sought.

### 2.5 GOR estimation and summery *OR*s

GOR methodology can be implemented by ORGGASMA software,^[11]^ which could obtain fixed-effect result and random-effect result. If *I*^2^ ≤ 25%, this was indicative of lack of significant heterogeneity among studies, and a fixed-effect result was adopted. If *I*^2^ ≥ 25%, it meant that significant heterogeneity existed among studies, and a random effect result was adopted. If GOR and its 95% confidence interval (CI) were >1, the G allele of rs266729 was likely a risk factor for T2DM; if the range of 95% CI contained 1, this indicated the likely absence of relationship between the G allele of rs266729 and T2DM; if GOR and its 95% CI were <1, this indicated that the G allele was probably a protective factor against T2DM.

Analysis for dominant ([GG + GC) vs. CC), recessive (GG vs. [GC + CC]) and additive (GG vs. GC, GC vs. CC) models was performed by STATA 12.0 (Stata Corp, College Station, TX), and the results were compared with those of GOR estimation.

#### 2.5.1 Genetic model estimation

The C allele of the rs266729 is a wild allele, whereas the G allele is a mutant. The *OR* for GC to CC was estimated as *OR*_1_, whereas that of GG to CC as *OR*_2_; the quotient of natural logarithm *OR*_1_ divided by natural logarithm *OR*_2_ was defined as parameter *λ* (Formula 1),^[12]^ wherein Log*OR*_2_ was the independent variable and Log*OR*_1_ was the dependent variable. The scatter plot was drawn in excel and the simple linear regression analysis was performed with Stata. Then *λ* and its 95% CI were used to determine the genetic model of the dominant G allele. If *λ* was 0, 1, or between 0 and 1, the corresponding genetic model of the G allele was recessive, dominant, or incompletely dominant, respectively; If *λ* >1 or <0, this indicated overdominance or underdominance. When *λ* = 0.5, it was called additive (a type of special incomplete dominance model).^[13]^ 
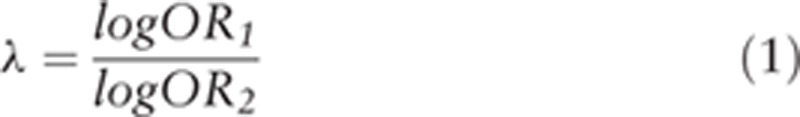


#### 2.5.2 Genetic effect estimation

The genetic effect estimation was verified by binary logistic regression analysis based on the genetic model in IBM SPSS Statistics 21.0 (SPSS Inc, Chicago, IL). If *λ* *=* 0, the genetic model was recessive, and the effect of GG would be 1, whereas those of GC and CC would be zero. With frequencies weighted, the genotypes, GG = 1, GC = 0, and CC = 0, were defined as categorical variables, whereas the groups (T2DM patients = 1 and control group = 0) were defined as binary dependent variables in the logistic regression, and the result was statistically significant. If *λ* *=* 1, the genetic model would be dominant, and the effects of GG and GC would be 1 and that of CC would be zero. If the genotypes, GG = 1, GC = 1, and CC = 0, were defined as categorical variables, and data were analyzed as the steps above, and the result was statistically significant. If *λ* was between 0 and 1, genetic model would be incompletely dominant, and genotypes were defined as categorical variables, and data were analyzed as the steps above. During the regression analysis, the CC genotype was taken as the reference category. Thus, if there were multiple relations between the GG and CC genotype coefficients, the genotypes were defined as ordinal variables and the data were analyzed as the steps above, and the result would be statistically significant (Table 1).

**Table 1:**
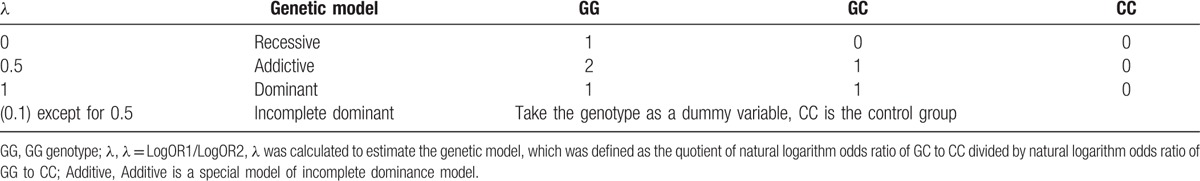
The values of genetic effect based on *λ*.

#### 2.5.3 Heterogeneity and publication bias test

The between-study heterogeneity was assessed by performing the Cochrane *χ*^2^-based *Q* test. If there was heterogeneity, meta regression was used to find out sources of heterogeneity. Additionally, the Begg funnel plot and Egger test were performed to determine whether the literature showed a publication bias. An asymmetric plot or *P* < .05 suggested possible publication bias. Sensitivity analysis was performed to assess the influence of each individual study by omitting one study at a time and calculating pooled *OR*s for the remaining studies. All statistical analyses above were performed by Stata.

## 3 Results

### 3.1 Articles included

**Figure 1:**
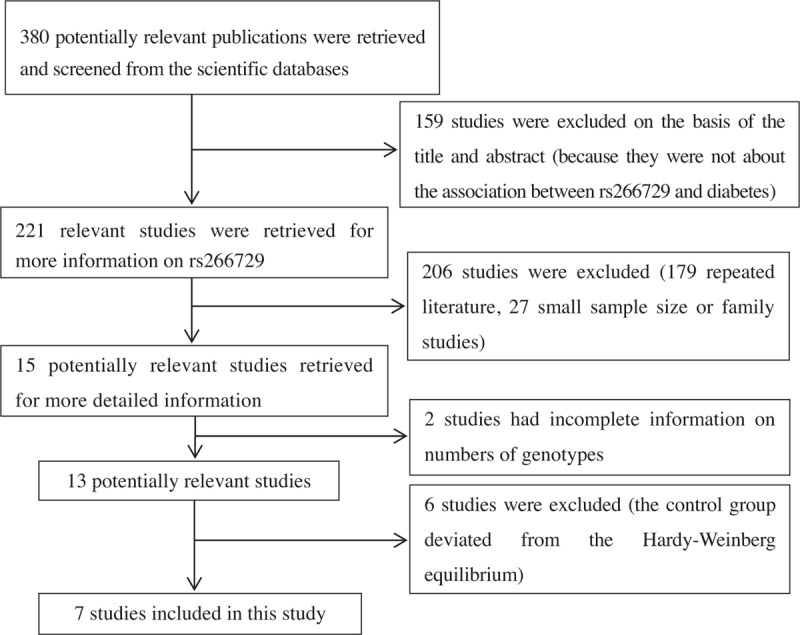
Schematic flow chart for selection of included studies (380 potentially relevant publications were retrieved and screened from the scientific databases, 7 literatures were included in this study.)

A total of 380 potentially relevant studies were retrieved and screened. Of these, 159 studies were not related to the association between the adiponectin rs266729 polymorphism and T2DM, and were excluded on the basis of title and abstract. Next, 206 studies were excluded because they did not meet the inclusion criteria, leaving 15 studies, from which an additional 2 literatures were excluded because they had incomplete information on the numbers of genotypes. Six studies were further excluded because they deviated from the HWE. Finally, 7 studies were included in this meta-analysis, including 1reported in Chinese and 6 reported in English (Fig. 1 Flow chart of paper selection).

### 3.2 Analysis results

#### 3.2.1 Characteristics of genotypes and quality assessment

The genotype distribution in the control groups of the 7 studies included was all within HWE. The total number of subjects was 12,323, comprising 5,948 cases and 6,395 controls. Mean (standard deviation) age of cases and controls was 59.50 (11.53) and 53.80 (11.65) years, respectively, whereas the proportion of males was 40.9% and 50.0%, respectively. The frequency of CC genotype in the case and control groups was 53.23% and 56.40%, respectively, whereas that of GC was 39.64% and 37.75%, respectively, and that of GG was 7.13% and 5.85%, respectively. The frequency of the G allele in the case and control groups was 26.95% and 24.73%, respectively. Among the 7 studies, 3 were on Asians, 2 were on Europeans, 1 was on Africans, and another was on Americans. The subjects in the remaining studies were Italian, French, Arabs, Indian, Chinese Han, whites. The characteristics of the study groups and individuals’ overall demographics are shown in Table 2. There were no significant statistical differences among the control populations of included studies, *χ*^2^ = 3.32, *P* = .77.

**Table 2:**
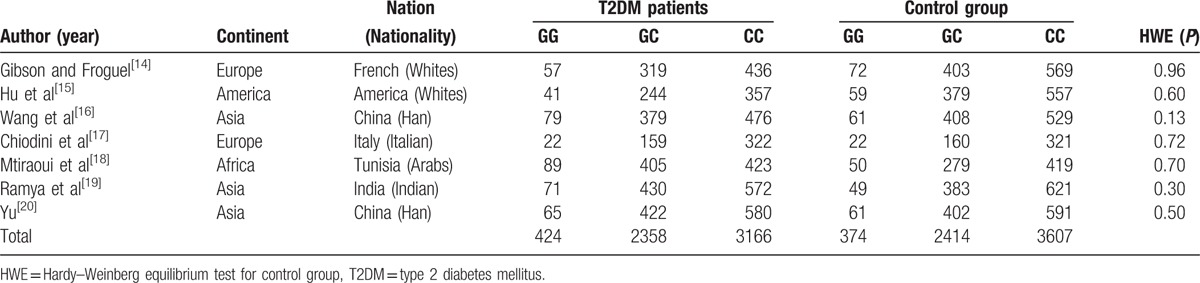
Characteristics of studies about the association between rs266729 alleles and T2DM.

Study quality scores are summarized in Table 3. Quality scores ranged from 7 to 9, with a median score of 8. The 7 studies included in this meta-analysis were all of high methodological quality.

**Table 3:**
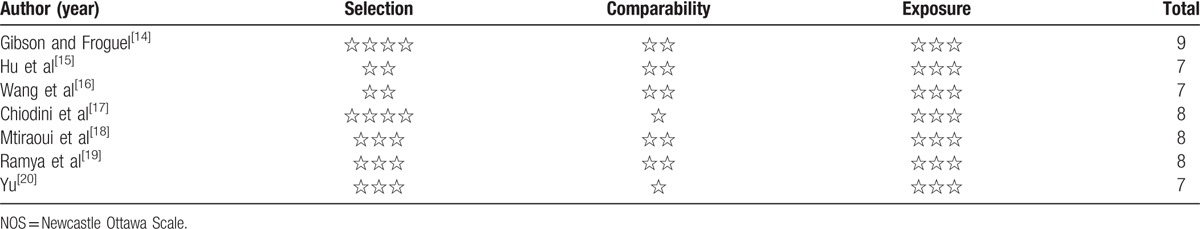
Methodological quality of included studies measured by NOS.

### 3.3 GOR value

**Figure 2:**
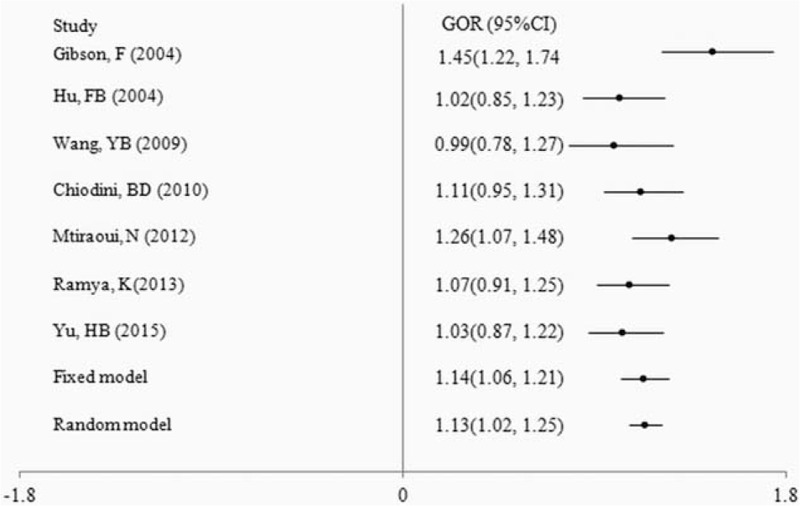
Forest plot of GOR estimation. Overall GOR (95%*CI*) was 1.12 (1.02, 1.22), I^2^ = 47.5%, *P* (heterogeneity) = 0.076). CI = confidence interval, GOR = generalized odds ratio.

GOR was used to efficiently estimate the association between the adiponectin rs266729 G allele and T2DM under unknown genetic model. The fixed-effect model result was: GOR = 1.14 and 95% CI (1.06, 1.21), whereas the random-effect result was: GOR = 1.13 and 95% CI (1.02, 1.25), *P* < .001. Because *I*^2^ = 55.32%, there was significant statistical heterogeneity among studies, and the random-effect result was adopted. Both GOR and 95% CI were >1, *P* < .001, indicating that adiponectin rs266729 G allele was a risk factor for T2DM. GOR forest plots are shown in Figure 2.

**Figure 3:**
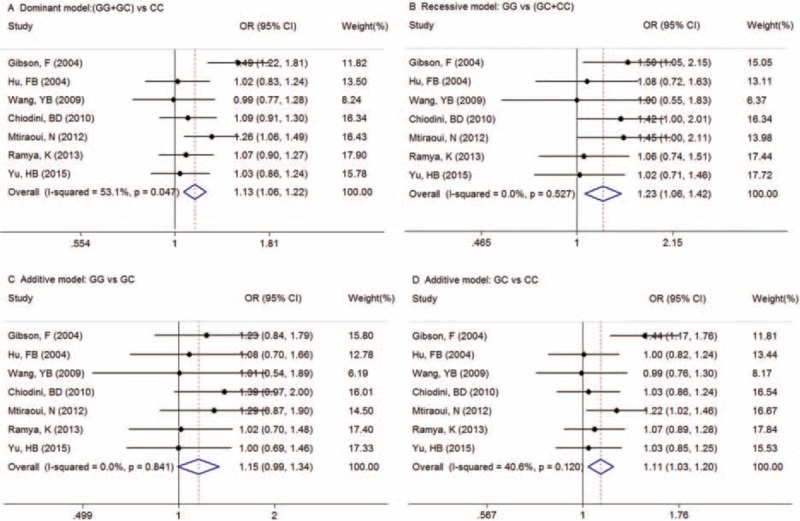
Forest plots of summary ORs using traditional analysis. OR (CI) of (GG + GC) vs. CC was 1.13 (1.06, 1.22); OR (CI) of (GG vs. [GC + CC]) was 1.23 (1.05, 1.46). GC vs. CC suggested G allele of rs266729 risk factor for T2DM, except of OR for GG to CC. CI = confidence interval, OR = odds ratio

Summary ORs are shown in Table 4, forest plots in Figure 3. There was tendency for an increased T2DM risk for (GG + GC) compared with CC (OR = 1.13, 95% CI [1.06, 1.22]*, P* *=* .001) and there was significant heterogeneity among studies (*P* = .047). (GC + CC) increased the risk for T2DM compared with GG (*OR* = 1.23, 95% CI [1.06, 1.42]*, P* *=* .005), whereas GC increased the risk compared with CC (OR = 1.11, 95% CI [1.03, 1.20]*, P* *=* .006), without significant heterogeneity among studies (*P* > .05). The risk of T2DM did not increase for GG compared with GC (OR = 1.15, 95% CI [0.99, 1.34]*, P* *=* .064).

**Table 4:**
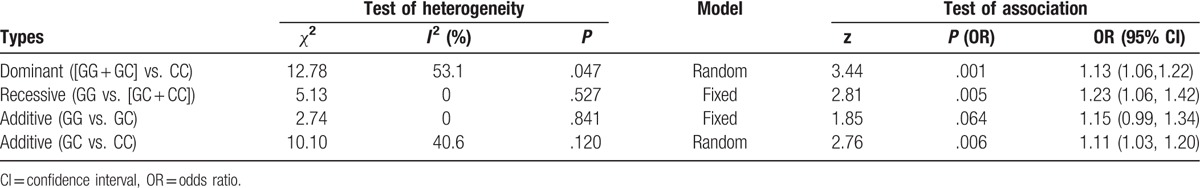
Summary ORs for genotype contrasts.

### 3.4 Genetic model estimation

**Figure 4:**
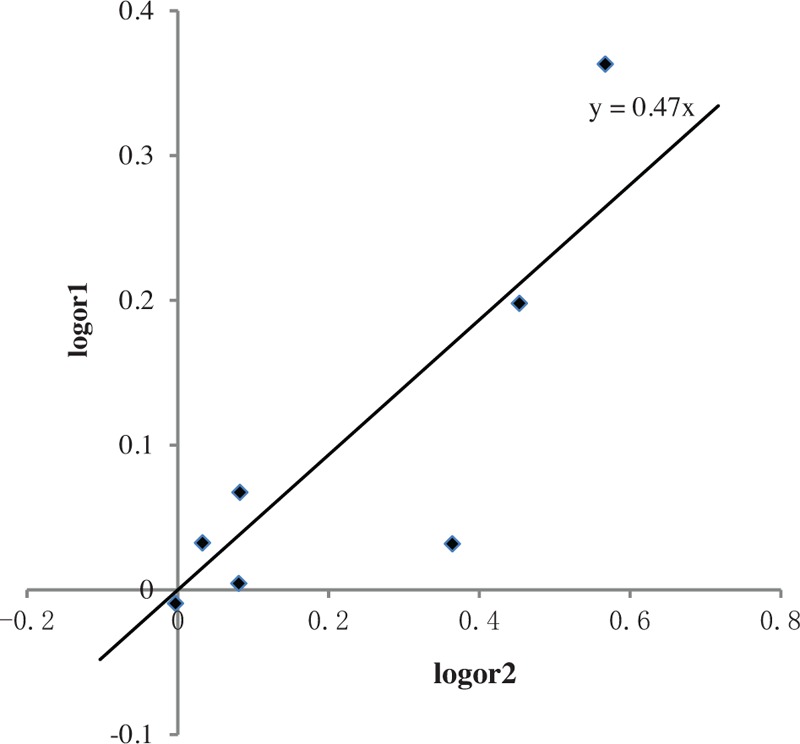
The genetic model of adiponectin rs266729 polymorphism (log*OR*2 represents horizontal ordinates, log*OR*1 represents vertical coordinates). The scatter plot showed that the equation was: y = 0.47x. Therefore *λ* = 0.47, the genetic model of the adiponectin rs266729 G allele was incomplete dominant.

The genetic model assessment of the G allele is given in Figure 4. In the simple linear regression and scatter diagram, the ratio of log*OR*_1_ and log*OR*_2_ was defined as *λ*, which was found to be 0.47 (0.29, 0.64). The genetic effect was estimated by binary logistic regression. The disease status was regarded as binary-dependent variable and the genotypes as categorical variables; the coefficient of the GC genotype was 0.11, whereas that of the GG genotype was 0.26 (*P* < .01). The GG coefficient was nearly twice as much as that of GC and the genotypes were GG = 2, GC = 1, and CC = 0, *λ* was 0.5, and the genetic model for the effect of the G allele on T2DM was additive. During the logistic regression analysis based on ordinal variable, the result showed that the genetic effect was 1.13 (1.06, 1.19) (Table 5).

**Table 5:**
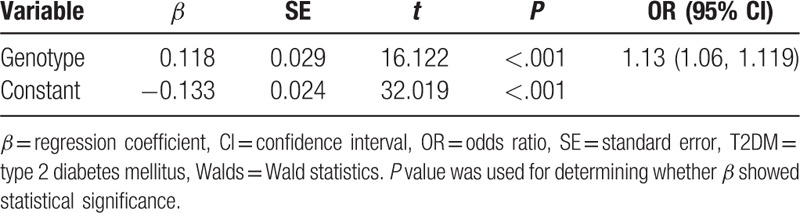
Genetic effect of rs266729 polymorphism on T2DM.

### 3.5 Heterogeneity and publication bias

**Figure 5:**
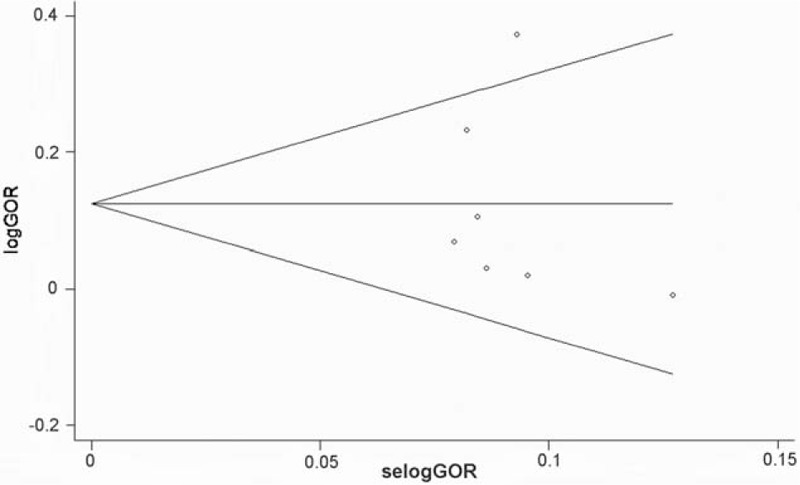
Begg funnel plot for rs266729 polymorphism (visual inspection of the funnel plots was symmetrical; the Begg test suggests there is no publication bias in this study.).

There existed heterogeneity among studies, *I*^2^ = 47.5%, *P* < .001. The meta-regression analysis showed that the sources of control were the reason for the between-study heterogeneity in Table 6, Tau^2^ < 0.001, adjusted *R*^2^ = 99.56%, *P* = .036. Visual inspection of the funnel plots indicated that it was symmetrical, and neither the Begg test nor the Egger test suggested publication bias. The *P* values of the Egger test were .48, whereas Begg funnel plot is shown in Figure 5. Results of sensitivity analysis showed that none of the individual studies dramatically influenced the pooled *OR*s, indicating that this meta-analysis had reliable and stable results.

**Table 6:**
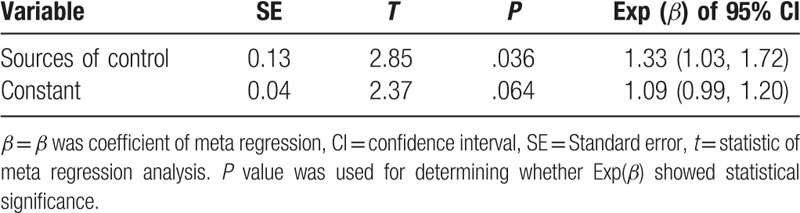
Sources of between-study heterogeneity.

## 4 Discussion

The mechanisms underlying the development of T2DM are complex. Nonetheless, adiponectin gene polymorphisms and abnormalities in plasma adiponectin levels have been associated with the disease. Accordingly, plasma adiponectin level closely correlates with IR and is decreased in T2DM patients. Studies also demonstrate a decline in adiponectin levels in clinical subjects and experimental animals with prediabetes. In line with plasma levels of adiponectin, the rs266729 polymorphism has been identified as a susceptibility locus for DM.^[21]^ However, studies of the association of the adiponectin rs266729 polymorphism with adiponectin level and DM often show inconsistent results. Some studies have suggested that the adiponectin rs266729 polymorphism was unlikely to influence T2DM in Europeans.^[17,22]^ In contrast, other studies have suggested that there was an association between the polymorphismand T2DM in Asians.^[7,19,23]^ The inherent genetic differences between ethnic group is to be responsible, just like the opposite trends of the calpain-10 rs3792267 allele and T2DM between the Uyghur and Chinese populations.^[24,25]^ In 2011, Han et al^[26]^ conducted a meta analysis to explore the association between SNPs of adiponectin gene and T2DM, and the results showed that the adiponectin rs266729 G allele was a risk factor for T2DM, with the pooled OR (95% CI) of 1.07 (1.03, 1.11, *P* = .001) for G versus C allele. However, the study did not provide the rs266729 genetic effect on the development of T2DM in different populations. Therefore, a more precise association between the adiponectin gene rs266729 polymorphism and T2DM risk reported in this meta-analysis for the first time is that the rs266729 G allele may increase the risk of T2DM in an additive genetic model.

In our study, the association between the rs266729 polymorphism and T2DM was evaluated objectively by analyzing GOR and its 95% CI under complete genotype distribution without a genetic model. The result showed that the rs266729 G allele was a risk factor for T2DM and people with the rs266729 G allele had 1.13-fold higher risk of developing T2DM compared to those without the G allele. Summery *ORs* ([GG + GC] vs. CC, GG vs. (GC + CC), GC vs. CC) were consistent with the result of GOR estimation, except for the OR of GG to GC. Then, the result of the genetic model assessment showed that the rs266729 G allele was incompletely dominant for inheritance of T2DM (*P* < .001), with *λ* of 0.47 (0.29, 0.64). Finally, the genetic effect was quantitatively evaluated by binary logistic regression, and the result further demonstrated that the genetic model of the rs266729 polymorphism was that of incomplete dominance: additive inheritance, *λ* = 0.5. Thus, the risk of developing DM increased by 13.0% (OR = 1.13) when the G allele was present at rs266729. Therefore, the rs266729 G allele was a risk factor for DM and the genetic model was that of additive inheritance.

The question, however, still remained regarding the molecular mechanisms of rs266729 polymorphism additively affecting T2DM. Sp1 is associated with the adiponectin gene promoter and its overexpression enhances the gene promoter activity.^[27]^ The rs266729 G allele alters the DNA sequence of the SP1-binding site of the transcriptional elements, resulting in a reduction in the transcriptional activity of the adiponectin gene promoter. Additionally, based on the location of DNA methylation in a genomic sequence, the consequence can produce varied genetic effects.^[28]^ For example, gene promoters and intergenic regions can become methylated and result in gene silencing,^[29]^ ultimately affecting the expression of genes during development.^[30–32]^ DNA methylation in the promoter region of rs266729 interacting with polymorphism may lead to the inhibited gene expression and low plasma adiponectin levels. These mechanisms can result in downregulation of the adiponectin gene resulting in decreased plasma adiponectin concentration. The deficiency or inactivity of adiponectin influences insulin sensitivity and accelerates the occurrence of T2DM.^[33,34]^ The pathophysiologic mechanisms of T2DM can be explained by IR and pancreatic *β*-cells failure,^[35]^ which closely relates to the adiponectin protein. Moreover, adiponectin plays a significant role in regulating glucose metabolism and IR.^[36,37]^ In addition, numerous studies report the association of the rs266729 G allele with low plasma adiponectin concentrations. Thus, having an appropriate level of adiponectin is essential to maintaining normal body metabolism.

Furthermore, between-study heterogeneity present in this study must be interpreted with caution; obviously, heterogeneity existed in healthy volunteers and passively selected populations. Other contributing factors may be the variable definition of T2DM patients and controls, genotype errors, and environmental history. There were no significant statistical differences (*P* > .05) among Asian, European, American, and African control populations; hence, there were no discrepancies in the genotype frequencies distribution of the rs266729 among control populations, and the genetic background of the rs266729 polymorphism for study populations was similar. The ethnicity could not influence the reliability of the results.

GWAS and meta-analyses have been used to evaluate the association between adiponectin gene polymorphism and T2DM,^[38,39]^ wherein OR values are collectively calculated by performing 2 separate pairwise comparisons or by assuming a specific underlying genetic model. Multicomparisons decline statistical power, increase type I error rate and false-positive rate.^[40]^ The effect of the rs266729 G allele on T2DM development could not be accurately estimated because of uncertain genetic model in previous studies. However, this study provides a genetic model-free approach to quantitatively analyze the association between the rs266729 polymorphism and T2DM. The genetic model was estimated on the basis of the genotype frequency available rather than assumed, whereas the genetic effects could be evaluated by binary logistic regression on the basis of genetic model. Furthermore, to guarantee the quality of this study, investigators used explicit criteria for inclusion and a strict procedure for data extraction. Similarly, the sample size of each included study was >500 in case and control, making the result more robust than previous meta-analysis that included studies with smaller sample sizes. In general, this method avoids the confounding of possible genetic models, making the results better powered and more informative than previous studies for the association between rs266729 polymorphism and T2DM. The result indicated that the rs266729 is a potential functional variant, which influences the adiponectin gene mRNA stability or adiponectin gene expression. It also indicates the importance of the rs266729 during the development of T2DM, which may provide important evidence to establish prevention and treatment strategies for T2DM. However, the limitation of this study was that original data could not be obtained, thereby limiting further evaluation of potential gene–gene interaction and gene–environment interaction.

In conclusion, the results of the present study reveal that the rs266729 in adiponectin gene is a susceptibility site for T2DM, and the genetic model of the G allele at this site contributing to the development of T2DM was that of incomplete dominance during inheritance. Individuals carrying the G allele can easily develop T2DM more than those without the G allele. However, these results highlight the challenge of polygene effect on T2DM. In the future, comprehensive interactions at the gene–gene and gene–environment level should be explored to further reveal the effect of multiple susceptibility genes on the development of T2DM.
